# BnaABF2, a bZIP transcription factor from rapeseed (*Brassica napus* L.), enhances drought and salt tolerance in transgenic *Arabidopsis*

**DOI:** 10.1186/s40529-016-0127-9

**Published:** 2016-06-01

**Authors:** Bi-Yan Zhao, Yu-Feng Hu, Juan-juan Li, Xuan Yao, Ke-de Liu

**Affiliations:** College of plant science and technology, Huazhong Agricultural University, Wuhan, 430070 China

**Keywords:** BnaABF2, bZIP transcription factor, Drought stress, Salt stress, Rapeseed

## Abstract

**Background:**

Abiotic stresses such as drought and salt stresses have a negative effect on the growth and productivity of plants. Improvement of stress tolerance through genetic engineering in plants has been reported in intense studies. Transcription factors play vital roles in plant adaptation to stresses by regulating expression of a great deal of target genes. A family of *Arabidopsis* basic region leucine zipper (bZIP) transcription factors that can recognize and bind to the abscisic acid (ABA)-responsive elements (ABREs) in promoter is named as ABRE binding factors (ABFs)/ABRE binding proteins (AREBs). They play a key role in the regulation of expression of downstream stress-responsive genes in ABA signalling. Genetic transformation of ABF/ABRE transcription factors has been suggested to be an effective approach for engineering stress-tolerant plants. However, whether the ABF/ABRE transcription factors are able to be used for generating stress-tolerant rapeseed plants has not yet been studied.

**Results:**

*BnaABF2*, encoding a bZIP transcription factor, was cloned from rapeseed in this study. Subcellular localization and transactivation analyses showed that BnaABF2 was localized to the nucleus with transactivation activity in plant cells. *BnaABF2* gene expression was induced by drought and salt stresses and BnaABF2 positively functions in ABA signalling during the vegetative stage. Overexpression of *BnaABF2* was found to render drought and salt tolerance to *Arabidopsis* plants. The resistance of the *BnaABF2*-expressing transgenic plants to drought and salt stresses is due to reduced water-loss rate and expression of stress-responsive genes such as *RD29B*, *RAB18* and *KIN2*. The expression of *RD29B*, *RAB18* and *KIN2* regulated by BnaABF2 is involved in an ABA-dependent stress signalling.

**Conclusions:**

Identification of the positive role of rapeseed BnaABF2 in plant tolerance to drought and salt provides evidence for ability of engineering stress-tolerant rapeseed plants by genetic transformation of BnaABF2.

**Electronic supplementary material:**

The online version of this article (doi:10.1186/s40529-016-0127-9) contains supplementary material, which is available to authorized users.

## Background

Drought and salt stresses are two major constraints adversely affecting plant growth and development, quality and productivity (Mittler 2006). To understand the molecular mechanism of plant adaptation to the stresses, an increasing number of studies have focused on cloning and characterization of signalling components in drought and salt stress signal transduction. These findings are basis of improvement of drought and salt tolerance through genetic engineering in plants. Transcription factors play pivotal roles in the response of plants to drought and salt stresses (Nakashima et al. 2009). They can bind to the *cis*-acting element in promoters of their target genes and regulate expression of downstream stress-responsive genes in drought and salt stress signal transduction (Zhu 2002; Yamaguchi-Shinozaki and Shinozaki 2006).

Under abiotic stress conditions, abscisic acid (ABA) accumulation is triggered and many stress-responsive genes are induced in plant adaptation to the stresses. By analyzing the promoters of ABA-induced genes, an important *cis*-element, the ABA-responsive element (ABRE, PyACGTGGC) was identified (Guiltinan et al. 1990; Mundy et al. 1990; Busk and Pagès 1998). Subsequent analyses revealed that multiple ABREs or an ABRE combined with a coupling element (CE) in promoters are required to regulate ABA-induced gene expression (Marcotte et al. 1989; Shen and Ho 1995; Shen et al. 1996; Hobo et al. 1999; Narusaka et al. 2003). In the *Arabidopsis* genome, nine basic region leucine zipper (bZIP) transcription factors, named as ABRE binding factors (ABFs)/ABRE binding proteins (AREBs), have been isolated by yeast one-hybrid screening using ABREs as bait (Choi et al. 2000; Uno et al. 2000). ABF/AREB family members are upregulated by various abiotic stresses, such as drought, salt, freezing and heat stresses and are involved in stress/ABA signalling in different plant species (Choi et al. 2000; Uno et al. 2000; Yáñez et al. 2009; Hossain et al. 2010; Huang et al. 2010).

In the *Arabidopsis* ABF/AREB family, *ABF1*, *ABF2/AREB1*, *ABF3* and *ABF4/AREB2* are highly induced by drought and salt stresses and by ABA in vegetative tissues (Choi et al. 2000; Uno et al. 2000; Fujita et al. 2005; Yoshida et al. 2015). Expression of stress- and ABA-responsive genes is inhibited in the *areb1 areb2 abf3* triple mutant in response to drought and salt stresses, resulting in increased sensitivity to drought stress (Yoshida et al. 2010). In contrast, overexpression of *ABF2*/*AREB1*, *ABF3* or *ABF4* involving in ABA signalling confers drought or/and salt tolerance to plants (Kang et al. 2002; Kim et al. 2004a; Fujita et al. 2005; Furihata et al. 2006). Therefore, *ABF2*/*AREB1*, *ABF3* and *ABF4*/*AREB2* are master transcription factors in ABA signalling and play cooperative roles in drought and salt stress tolerance (Kang et al. 2002; Kim et al. 2004b; Fujita et al. 2005; Furihata et al. 2006; Yoshida et al. 2010). Recent study has demonstrated that the *abf1 areb1 abf3 areb2* mutants exhibit elevated sensitivity in response to drought stress and reduced ABA sensitivity in primary root growth compared with the *areb1 abf3 areb2* mutant (Yoshida et al. 2015). These studies suggest ABF1, ABF2/AREB1, ABF3 and ABF4/AREB2 are functionally redundant in *Arabidopsis*. In early ABA signalling, ABA binds to pyrabactin resistance 1/PYR1-like receptors (PYR/PYL/RCARs), which in turn bind to and inactivate type 2C protein phosphatases (PP2Cs) to remove the repression by activating PP2Cs of SNF1-type kinases (SnRK2s) (Fujii et al. 2009; Miyazono et al. 2009; Nishimura et al. 2009). SRK2D/SnRK2.2, SRK2E/SnRK2.6/OST1 and SRK2I/SnRK2.3 co-localize and interact with the ABF/AREBs in plant cell nuclei (Fujita et al. 2009; Yoshida et al. 2010, 2015). ABF/AREBs are phosphorylated by the SnRK2s (Uno et al. 2000; Furihata et al. 2006; Fujii et al. 2009) to regulate downstream gene expression in ABA signalling.

Although the ABF/AREBs transcription factors have been comprehensively studied in *Arabidopsis*, we have little knowledge of their orthologs in rapeseed (*Brassica napus*). Recently, *BnaABF1*, *BnaABF3*, *BnaABF4*, *BnaAREB3* and *BnaABI5* have been cloned and identified from rapeseed by expressed sequence tag (EST) analyses to test the interaction with the identified calcium-dependent protein kinases (CPKs) in vitro (Zhang et al. 2014). Here, we cloned *BnaABF2* from rapeseed and characterized its positive role in drought and salt resistance. The introduction of *BnaABF2* conferred drought and salt stress tolerance in transgenic *Arabidopsis* plants. This study suggests that genetic transformation of *BnaABF2* is likely used for engineering stress tolerance in rapeseed.

## Methods

### Plant materials and growth conditions

Transgenic *Arabidopsis* plants were generated in a wild-type (WT) ecotype Columbia (Col-0) background in this study. Seeds were surface sterilized and then sown on MS medium (Murashige and Skoog 1962) containing 0.8 % (w/v) agar and 1 % (w/v) sucrose. The sown seeds were vernalized for 2 days in the dark at 4 °C and all plants were grown in a growth chamber under a 16 h light/8 h dark photoperiod and 70 % humidity at 23 °C.

### Plasmid constructions and generation of transgenic plants

In order to generate transgenic *Arabidopsis* plants ectopically overexpressing *BnaABF2*, a 1.1 kb full-length cDNA of *BnaABF2* gene was amplified from a cDNA of *B. napus* cv. Westar by RT-PCR and inserted into the *Bam*HI/*Sma*I cloning sites of BarII-pUBQ10-MCS driven by the *Arabidopsis* ubiquitin-10 gene promoter. To analyze cellular localization of BnaABF2, the plasmid pGFP-BnaABF2 was constructed by cloning the cDNA fragment of *BnaABF2* into the *Spe*I/*Xho*I cloning sites of vector BarII-pUBQ10-GFPn. For transactivation assay, the plasmid pGBKT7- BnaABF2 was made by inserting the cDNA fragment of *BnaABF2* into the *Eco*RI/*Pst*I cloning sites of vector pGBKT7 (Clontech). The sequences of primers used for plasmids constructions are listed in Additional file 1: Table S1. *Agrobacterium tumefaciens* strain GV3101 harboring the plasmid BarII-pUBQ10-BnaABF2 was used to transform with Col-0 plants by floral dip-mediated infiltration (Clough and Bent 1998) to generate *Arabidopsis* transgenic plants overexpressing *BnaABF2*.

### RT-PCR analyses

Semi-quantitative RT-PCR was performed to analyze *BnaABF2* (HE616526) gene expression. Total RNA was isolated from leaves of 2-week-old plants using an RNAprep Pure Plant kit (BioTeKe, China), according to manufacturer’s instructions. RNA samples were reverse-transcribed with a ReverAid Fist Strand cDNA synthesis kit supplemented with RNase-free DNase I set (Thermo Scientific, USA). The expression level of the *ACTIN2* gene (AT3G18780) was used as a loading control. Quantitative RT-PCR was performed to assess expression levels of responsive genes under normal or drought and salt stress. The cDNA was amplified using a SYBR Green master mixture (Bio-Red, USA) with a LightCycler 96 (Roche, USA). *β*-*ACTIN8* (AT1G49240) gene expression was used as an internal control. The sequences of primers used for RT-PCR analyses are listed in Additional file 1: Table S2.

### Drought and salt stress treatments, stomatal aperture bioassay and water-loss assay

For drought stress treatment, 2-week-old WT and transgenic plants grown in soil were deprived from water for 2 weeks and rewatered. For salt stress treatment, 2-week-old plants grown in soil were irrigated with 300 mM NaCl every other day for 2 weeks. Stomatal aperture bioassay was carried out as described previously by Zhang et al. (2001). Leaves from 4-week-old *Arabidopsis* plants were floated in buffer containing 10 mM KCl, 50 μM CaCl_2_ and 10 mM MES/KOH (pH 6.15). After induced stomatal opening in the light for 3 h, the leaves were treated with different concentration of ABA for 3 h to assay ABA-induced stomatal closure. For water-loss quantification, detached rosette leaves from 4-week-old plants were placed on filter paper at room temperature. Water loss was monitored at the indicated times and calculated as the percentage of initial fresh weight (% FW).

### Subcellular localization analysis and transactivation assay

Transient expression experiment was performed to analyze the subcellular localization of BnaABF2 as described previously by Voinnet et al. (2003). The transformed Agrobacterium cells GV3101 carrying with the plasmid pGFP-BnaABF2 were harvested and resuspended in 10 mM MES-KOH, pH 5.6, containing 10 mM MgCl_2_ and 150 mM acetosyringone, and then mixed with Agrobacterium suspension of p19 expressing the silencing suppressor to an OD_600_ of 0.8. The mixed suspension was injected into expanded leaves of tobacco plants (*Nicotiana benthamiana* cv. SR1) until they were 4 weeks old. The injected leaves were observed after 3 days with a laser scanning confocal imaging system (TCS SP2, Leica).

For transcriptional activation assay, the plasmid pGBKT7-BnaABF2 was transformed into *Saccharomyces cerevisiae* strain AH109 according to manufacturer’s instructions (Matchmaker™ GAL4 Two-Hybrid System 3, Clontech). The transformants were plated onto SD/-Trp medium for positive selection. The positive colonies were then cultured onto SD/-Ade/-His/-Trp medium supplemented with 20 μg/ml X-α-Gal to examine MEL1 encoded α-galactosidase activity.

## Results

### Cloning of rapeseed *BnaABF2* gene and analysis of BnaABF2 protein sequence

**Fig. 1:**
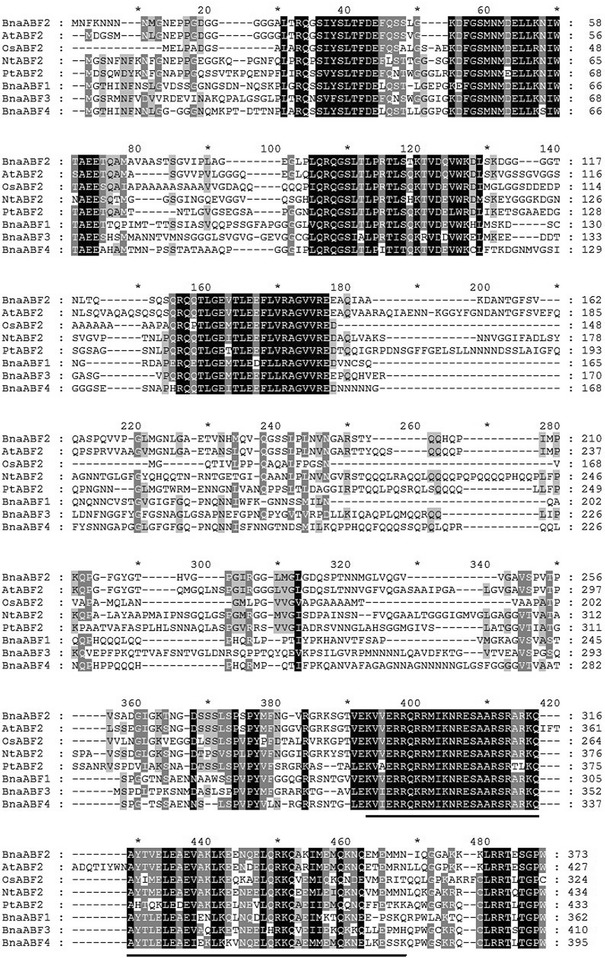
Multiple alignment of the deduced amino acid sequence of BnaABF2 and its homologs. The alignment was built by using ClustalX 1.81 and GeneDoc 3.2 tools. Identical and similar amino acids were shaded in *black* and *gray*, respectively. The basic-leucine zipper *bZIP* region was shown by *black line.* BnaABF2 (CCE88374), BnaABF1 (AGG35955), BnaABF3 (AGG35956) and BnaABF4 (AGG35957) are from rapeseed (*Brassica napus*). AtABF2 (NP_001185157) is from *Arabidopsis thaliana*. OsABF2 (DI201490) is from *Oryza sativa*. NtABF2 (AHD24943) is from *Nicotiana tobacum*. PtABF2 (ABN58425) is from *Populus trichocarpa*

**Fig. 2:**
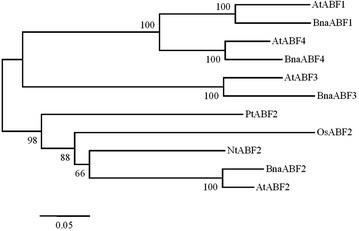
A phylogenetic tree constructed based on the amino acid sequences of BnaABF2 and other 10 ABFs, including BnaABF1, BnaABF3, BnaABF4, AtABF1 (NP_001185183), AtABF2, AtABF3 (NP_849490), AtABF4 (NP_001189934), OsABF2, NtABF2 and PtABF2. The numbers beside the branches indicate bootstrap values from 1000 replications. The relative amount of change along branches is indicated by the *scale bar*

A 1122 bp fragment containing a complete coding region was obtained from rapeseed (*Brassica napus* cv. Westar) by reverse transcription PCR. This gene encoding a predicted polypeptide of 373 amino acids was designated as *BnaABF2* (HE616526). Multiple alignments between BnaABF2 and seven other ABF proteins revealed a high level of sequence similarity among each other (Fig. 1). At the C-terminus of BnaABF2 protein there was a 65-amino acid basic region leucine zipper (bZIP) conserved domain (from position 292 to 356, E-value 3.94e-13) (http://smart.embl-heidelberg.de/smart/show_motifs.pl) (Fig. 1). The phylogenetic tree constructed based on amino acid sequences of BnaABF2 and other 10 ABFs from different plant species showed that BnaABF2 was most closely related to *Arabidopsis* ABF2/AREB1, while it was most distant to *Arabidopsis* ABF1 (Fig. 2). The phylogenetic analysis together with the conserved domain analysis will facilitate further study of the function of BnaABF2.

### Rapeseed *BnaABF2* gene expression was induced by drought and salt stresses

**Fig. 3:**
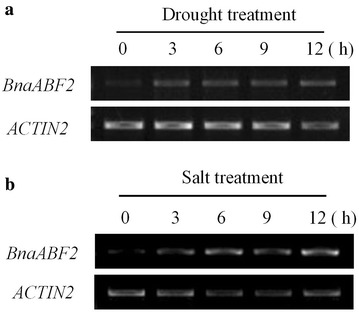
Semi-quantitative RT-PCR analyses of *BnaABF2* expression under drought and salt stresses in rapeseed. Leaves of 4-week-old rapeseed plants were subjected to drought condition (**a**) and 200 mM NaCl for salt treatments (**b**) for 0, 3, 6, 9 and 12 h

Previous studies have revealed that ABF2/AREB1, the closest homologue of BnaABF2, can enhance drought and salt resistances in *Arabidopsis* (Kim et al. 2004b; Fujita et al. 2005). To investigate whether the expression of *BnaABF2* was altered by drought and salt stresses, the leaves of 2-week-old plant were subjected to drought and salt treatments. Semi-quantitative RT-PCR analysis showed that *BnaABF2* gene expression was induced by the stresses (Fig. 3). The increased expression of *BnaABF2* under drought and salt stresses implies that BnaABF2 may be involved in plant adaptation to the stresses, like *Arabidopsis* ABF2/AREB1.

### Overexpression of *BnaABF2* conferred drought and salt tolerance to *Arabidopsis* plants

**Fig. 4:**
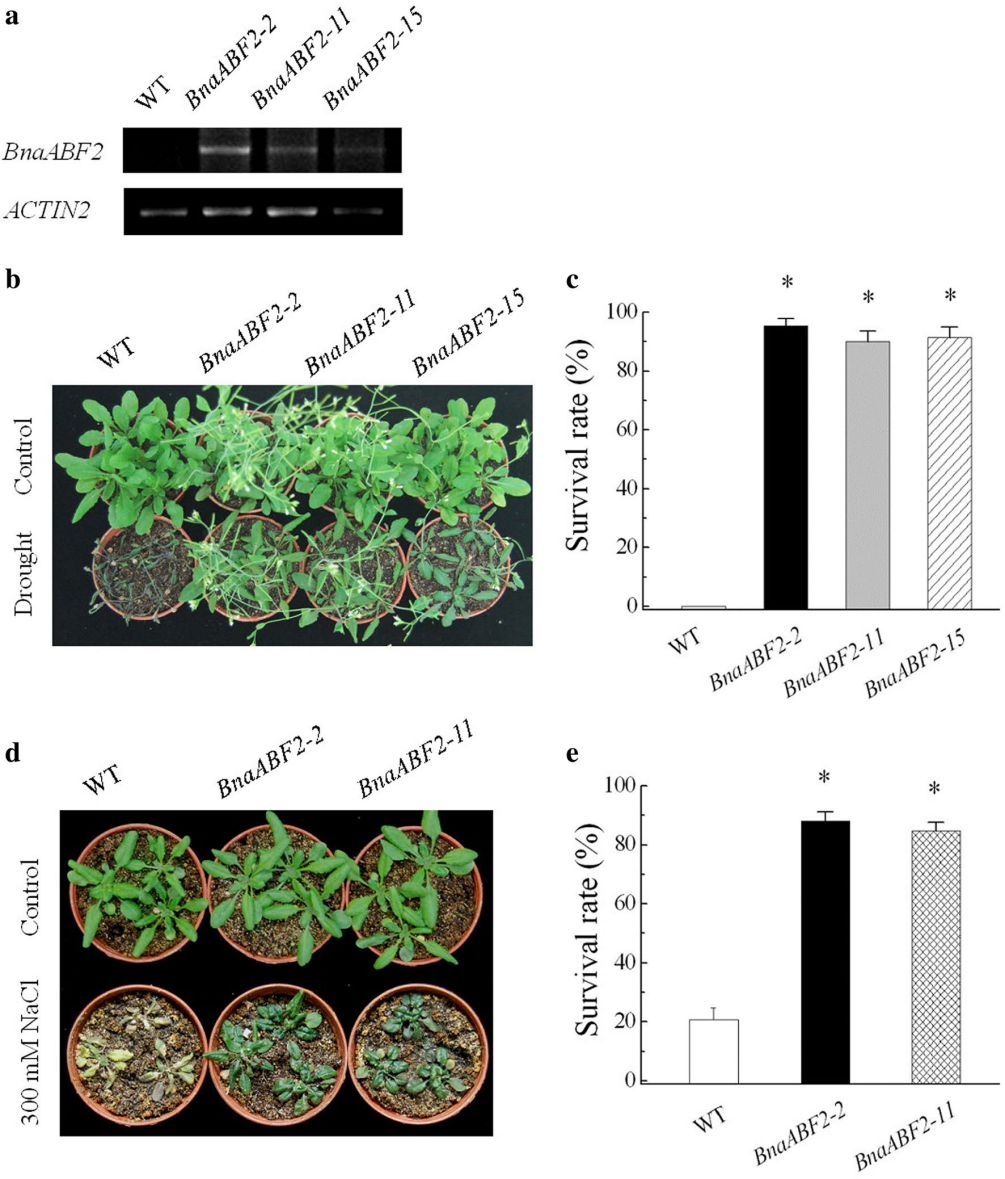
Responses of wild type *WT* and transgenic *Arabidopsis* plants expressing *BnaABF2* to drought and salt stresses. **a** Expression levels of *BnaABF2* gene in WT, BnaABF2-2, BnaABF2-11 and BnaABF2-15 transgenic lines. *ACTIN2* was used as a loading control. **b** Two-week-old WT and transgenic plants were well watered (Control) or deprived of water for 2 weeks and then rewatered (Drought). The photos were taken on day 1 after rewatering. **c** Survival rates of 4-week-old WT and transgenic plants on day 1 after rewatering. **d** Two-week-old WT and transgenic plants grown in soil under normal conditions (Control) or following salt treatment for 2 weeks (300 mM NaCl). **e** Survival rates of 4-week-old WT and transgenic plants after salt stress in soil. Values are mean ± SE from three independent experiments (n = 50). *, P < 0.01 compared with WT in the same treatment

**Fig. 5:**
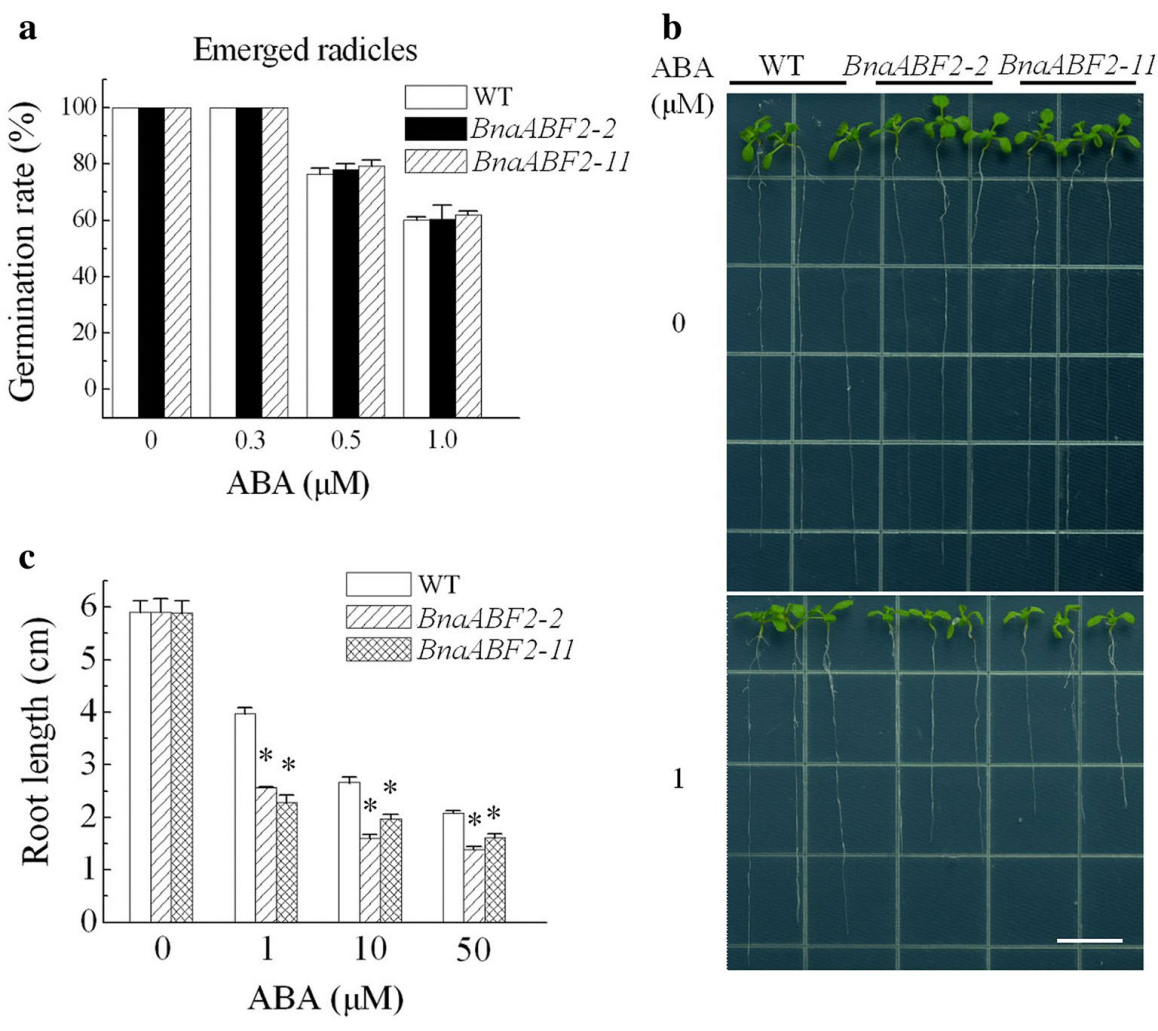
Responses of WT and the transgenic plants to ABA. **a** Seeds were sown on MS medium supplemented with 0, 0.3, 0.5 or 1.0 μM ABA. Germination rates (%) were analyzed at day 2 after stratification. Values are mean ± SE from three independent experiments (n > 50 for each experiment). **b** Seedlings grew 7 days after transferred to MS medium supplemented with 0, 1, 10 or 50 μM ABA. Seedlings were transferred from ABA-free medium to ABA-containing medium 4 days after stratification. *Scale bar* = 1 cm. **c** Primary root lengths were assayed at day 7 after transfer. Values are the mean ± SE of three independent experiments (n = 30). *P < 0.05 compared with WT control

In order to study the function of BnaABF2, we generated transgenic *Arabidopsis* plants expressing *BnaABF2*. Twenty-two positive lines were identified and semi-quantitative RT-PCR was performed to determine the expression levels of *BnaABF2* in T_3_ homozygous transgenic plants. *BnaABF2* were stably expressed in transgenic lines but were not expressed in WT plants (Fig. 4a). Three lines, BnaABF2-2, BnaABF2-11 and BnaABF2-15 were chosen for further analyses. Drought stress was imposed on 2-week-old WT and transgenic plants by withholding water for 14 days and then the plants were rewatered. The results showed that the three transgenic plants expressing *BnaABF2* could recover from wilting by rehydration in contrast to WT plants (Fig. 4b). After rewatering for 1 day, for example, ~95 % (92.9–97.7 %) of BnaABF2-2 transgenic plants recovered, whereas all the WT plants wilted and died (Fig. 4c). For salt stress treatment, 2-week-old WT and transgenic plants were watered with 300 mM NaCl solution for 14 days. ~79 % (75.3–83.4 %) of WT wilted and died; on the contrary, more than 85 % transgenic plants survived (Fig. 4d, e). These results suggest that the introduction of *BnaABF2* from rapeseed confers drought and salt tolerance to the *Arabidopsis* plants. In addition, none of the transgenic lines showed any obvious difference in abscisic acid (ABA) sensitivity in comparision to WT plants by scoring the emerged radicles during the seed germination (Fig. 5a). To assess the ABA response of the transgenic plants during the vegetative stage, 4-day-old seedlings were transferred from the ABA-free medium to MS medium containing 0, 1, 10 or 50 μM ABA. The length of the primary root was measured 1 week later. The primary root growth of the transgenic plants was significantly more inhibited by ABA when compared with that of WT plants (Fig. 5b, c), showing the increased ABA sensitivity of the transgenic plants expressing *BnaABF2*. These data suggest that BnaABF2 functions in ABA signalling during the vegetative stage, consistent with the previous studies on ABFs in *Arabidopsis* (Yoshida et al. 2010).

### Overexpression of *BnaABF2* enhanced stomatal sensitivity to ABA and capacity to conserve water

**Fig. 6:**
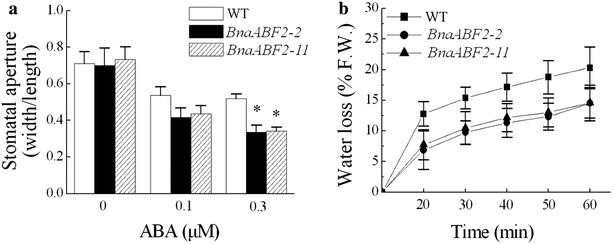
ABA-induced Stomatal closure in WT and the transgenic plants. **a** Stomatal closure assays following the treatments of 0, 0.3 or 0.5 μM ABA. Values are mean ± SE from three independent experiments (n = 50). *P < 0.05 compared with WT in the same treatment. **b** Transpirational water loss from detached leaves of 4-week-old WT and transgenic plants at the indicated time points. The data are shown as the mean ± SD from six individual plants per genotype

In order to assess whether the enhanced drought and salt stress tolerance is caused by altered stomatal aperture and transpiration rates, we measured ABA-induced stomatal closure and water-loss rates of the transgenic plants. After 3 h of ABA treatment (0.1 or 0.3 μM), the transgenic plants expressing *BnaABF2* showed an increased sensitivity to ABA in stomatal closure compared with WT plants (Fig. 6a). Transpirational water loss of detached rosette leaves was determined from 4-week-old WT and transgenic plants at room temperature with a humidity of 40–50 %. Reduced water-loss rates were found in the transgenic plants in comparision to WT plants (Fig. 6b). These present data suggest that the enhanced resistance of the *BnaABF2*-expressing transgenic plants to drought and salt stresses is a consequence of reduced stomatal aperture and water-loss rates.

### BnaABF2 was localized to the nucleus and had transactivation activity

**Fig. 7:**
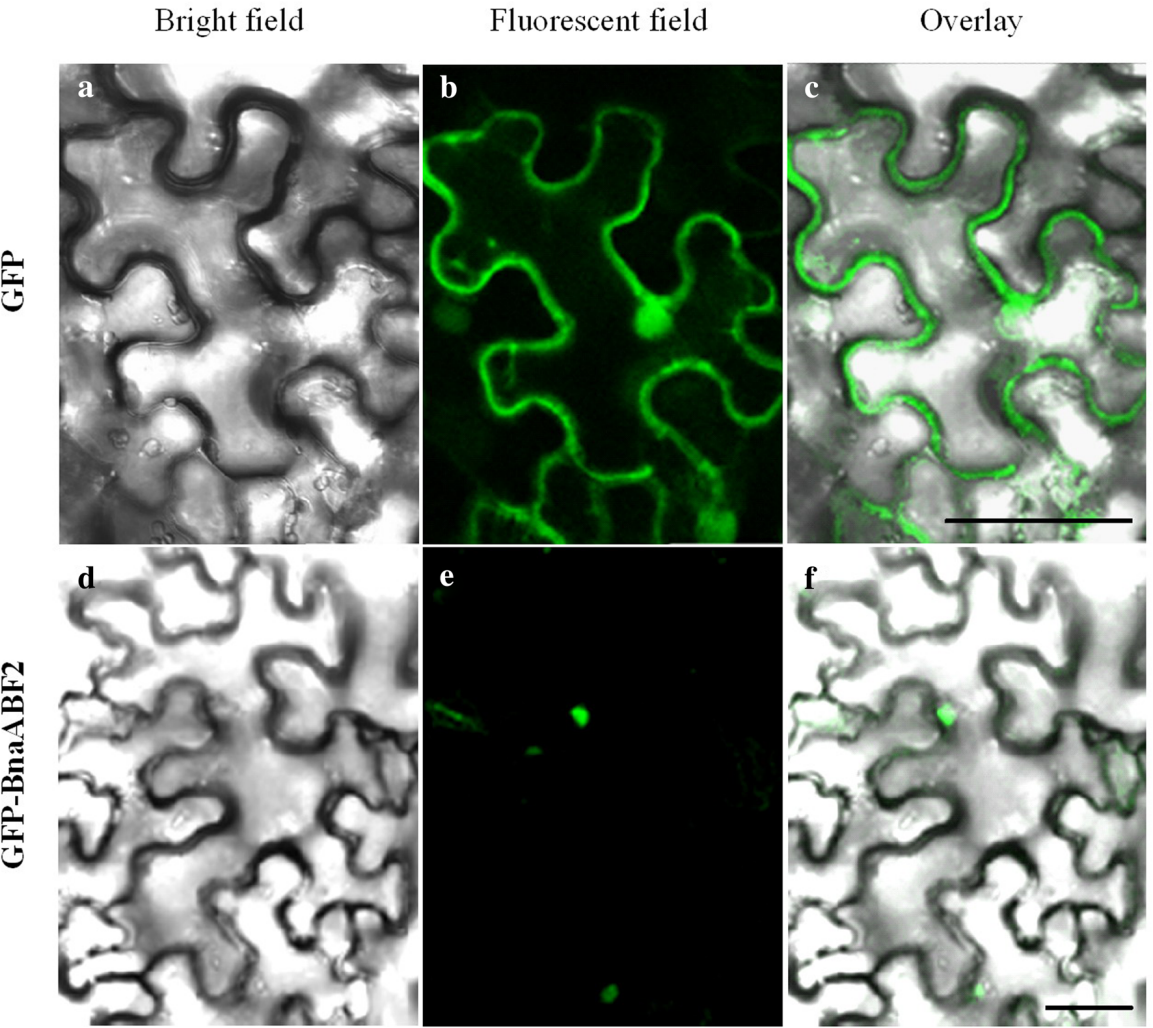
Subcellular localization of GFP-BnaABF2 fusion protein in leaf epidermal cells of tobacco. GFP-BnaABF2 plasmid and vector control (GFP) were transformed into tobacco epidermal cells by agroinfiltration, respectively. Bright-field images (**a**, **d**), fluorescent-field images (**b**, **e**), and the merged images (**c**, **f**) of leaf epidermal cells expressing GFP (**a**, **b**, **c**) or GFP-BnaABF2 fusion protein (**d**, **e**, **f**). *Bar*, 50 μm (**c**, **f**)

Protein sequence analysis showed that BnaABF2 was a putative leucine zipper transcription factor with a conserved bZIP domain (292–356 aa) (Fig. 1), implying that BnaABF2 may be localized to the nucleus. In order to analyze subcellular localization of BnaABF2, GFP-BnaABF2 fusion construct and 35S-GFP vector control were transiently expressed in leaf epidermal cells by tobacco agroinfiltration. When GFP plasmid was transformed, green fluorescence signals were observed in the whole cell (Fig. 7a–c). In contrast, fluorescence was exclusively detected in nuclei of leaf epidermal cells expressing the GFP-BnaABF2 fusion plasmids (Fig. 7d–f). These results suggest that BnaABF2 is a nuclear protein.

**Fig. 8:**
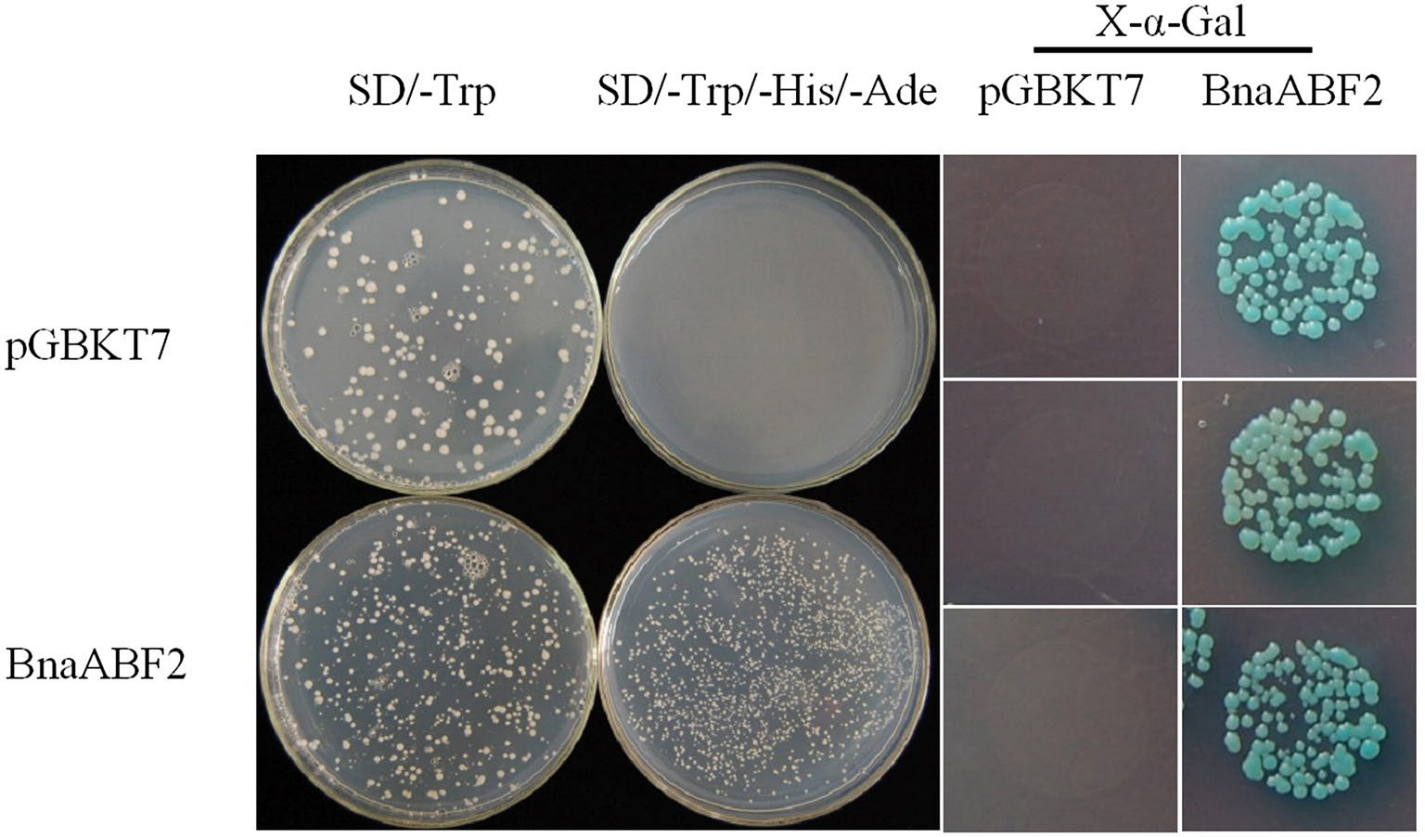
Analysis of transactivation activity of BnaABF2. The AH109 competent yeast cells were transformed with either control vector (pGBKT7) or pGBKT7-BnaABF2 plasmids and were streaked on SD/-Trp, SD/-Trp/-His/-Ade medium plate, or SD/-Trp/-His/-Ade supplemented with 20 μg/ml X-α-Gal medium plates

The nuclear localization of BnaABF2 promoted us to assay whether BnaABF2 has transactivation activity with a yeast two-hybrid system. The full-length coding region of BnaABF2 was fused to the DNA-binding domain of GAL4 to generate pGBKT7-BnaABF2 plasmids. pGBKT7-BnaABF2 and pGBKT7 control plasmids were then transformed into the AH109 competent yeast cells, respectively. The yeast cells carrying either pGBKT7 or pGBKT7-BnaABF2 plasmids and were streaked on SD/-Trp (Fig. 8). The yeast colony was cultured and streaked on SD/-Trp/-His/-Ade medium plate. On the SD/-Trp/-His/-Ade medium, the yeast cells transformed with the pGBKT7 control plasmid could not grow, whereas those transformed with the pGBKT7-BnaABF2 plasmid grew normally (Fig. 8), suggesting BnaABF2 functions as a transcriptional activator. In addition, when cultured on SD/-Trp/-His/-Ade medium supplemented with 20 μg/ml X-α-Gal plates, only the yeast cells harboring pGBKT7-BnaABF2 turned blue (Fig. 8). All the results suggest that BnaABF2 has transactivation activity in yeast.

### Transcriptional alteration of responsive genes after drought and salt stresses

**Fig. 9:**
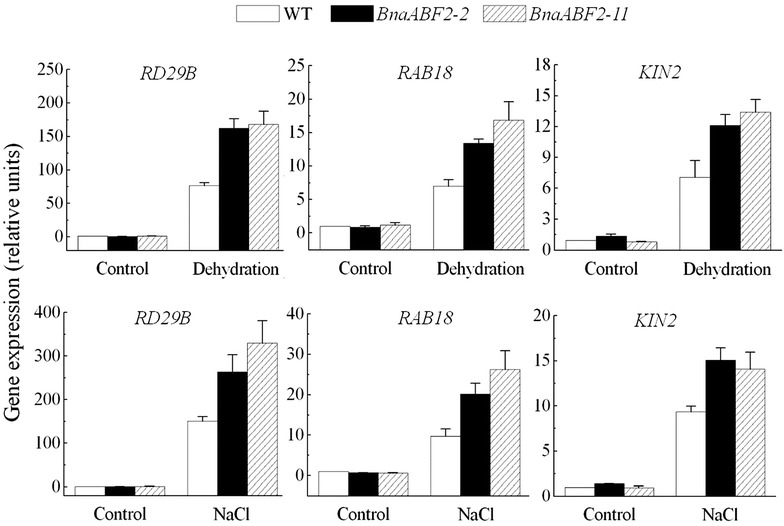
Expression levels of some drought- and salt-responsive genes in WT and *BnaABF2*-expressing transgenic plants under drought or salt stress condition. Two-week-old plants grown on the MS medium supplemented with 1 % sucrose were treated with dehydration or 200 mM NaCl for 6 h, respectively, while the control plants were not subjected to the stresses. The expression levels are presented as relative units with the levels of non-treated WT plants being taken as 1. Values are the mean ± SE of three independent experiments

Quantitative RT-PCR was performed to test the expression of some drought- and salt-responsive genes in ABA-dependent signal transduction such as *RD29B* (Yamaguchi-Shinozaki and Shinozaki 1994), *RAB18* (Lang and Palva 1992) and *KIN2* (Gilmour et al. 1992; Kurkela and Borg-Franck 1992) after the plants had been subjected to dehydration or salt treatment for 6 h, respectively (Fig. 9). Overexpression of *BnaABF2* did not affect the expression levels of *RD29B*, *RAB18* and *KIN2* assayed in the absence of stresses, whereas the expression levels of *RD29B*, *RAB18* and *KIN2* assayed in the presence of stresses were significantly increased in the transgenic plants expressing *BnaABF2* in comparison with the expression levels in the WT plants (Fig. 9). These results indicate that the adaptive processes to drought and salt stresses in the *BnaABF2*-expressing transgenic plants may be regulated by downstream responsive gene expression in ABA signal transduction.

## Discussion

Rapeseed (*Brassica napus* L.) is one of the main oil crops in the world. The global rapeseed production in 2013 reached 72.7 million tonnes (FAO 2013; http://faostat.fao.org/). Its cultivation is not only for edible vegetable oil, but also for industrial materials including livestock meal, lubricants and biodiesel. Rapeseed (A_n_A_n_C_n_C_n_, 2n = 38) was formed into an allotrtraploid crop species around 7500 years ago by natural hybridization between *Brassica rapa* (AA, 2n = 20) and *Brassica oleracea* (CC, 2n = 18), followed by chromosome doubling (Nagaharu 1935; Ziolkowski et al. 2006). BnaA10g28780D and BnaC06g00420D, two orthologs of *Arabidopsis* *ABF2/AREB1* (NM_001198228) gene, were found by searching the genomic database of rapeseed (Chalhoub et al. 2014). It should be noted that only the expression of BnaA10g28780D (*BnaABF2*) from A_n_ subgenome was induced by drought and salt stresses (Fig. 3) whereas the expression of BnaC06g00420D from C_n_ subgenome was not altered by the stresses (data not shown), implying the functional differentiation of these two genes. Therefore, BnaA10g28780D (*BnaABF2*) was used to generate the transgenic *Arabidopsis* plant expressing rapeseed *ABF2*. In this study, the transgenic plants expressing *BnaABF2* was found to be tolerant to drought and salt stresses (Fig. 4). These results are consistent with the previous findings that the homolog ABF2/AREB1 (Fig. 2) plays positive role in drought and salt tolerance in *Arabidopsis* (Kang et al. 2002; Kim et al. 2004b; Fujita et al. 2005; Furihata et al. 2006; Yoshida et al. 2010). This is to our knowledge the first study that characterizes the physiological roles of BnaABF2 in plants.

ABA synthesis is stimulated by drought and salt stresses and ABA participates in both physiological behaviors such as stomatal movement and photosynthetic regulation (quick processes) and gene expression modification (slow processes) in response to the stresses in plants. ABA-induced stomatal closure facilitates the reduction of transpiration and water loss, relieving the damage derived from the stresses in plants (Zhu 2002; Yamaguchi-Shinozaki and Shinozaki 2006). As shown in Figs. 5c and 6a, overexpression of *BnaABF2* enhanced ABA responsiveness in seedling growth and stomatal closure, suggesting BnaABF2 plays a positive role in ABA signalling during the vegetative stage. BnaABF2 is likely to trigger stomatal closure to avoid water loss under drought and salt conditions (Fig. 6b).

ABA stimulates expression of transcriptional activators such as ABF/AREB transcription factors, which are responsible for activating expression of downstream stress-responsive genes in ABA-dependent stress signalling (Zhu 2002; Yamaguchi-Shinozaki and Shinozaki 2006). As shown in Figs. 1 and 2, BnaABF2, the closest homolog of *ABF2/AREB1*, also contains a bZIP conserved domain, implying BnaABF2 is a putative bZIP transcription factor in rapeseed. Furthermore, the findings of nuclear subcellular localization (Fig. 7) and transactivation activity (Fig. 8) provide evidences for BnaABF2 is a transcription factor. To understand the molecular mechanisms of the stress tolerance in transgenic plants expressing *BnaABF2*, quantitative RT-PCR was performed to detect the expression levels of responsive genes in response to drought and salt stresses. Three late embryogenesis abundant (LEA) class proteins (Battaglia et al. 2008; Bies-Ethève et al. 2008), RD29B (Nordin et al. 1993; Yamaguchi-Shinozaki and Shinozaki 1994), RAB18 (Lang and Palva 1992), and KIN2 (Gilmour et al. 1992; Kurkela and Borg-Franck 1992), play crucial roles in ABA-dependent stress tolerance in *Arabidopsis*. It is noteworthy that all these genes carry at least two ABRE motifs, which is required for transcriptional activation by ABF/AREB transcription factors (Choi et al. 2000; Uno et al. 2000). The expression levels of *RD29B*, *RAB18* and *KIN2* in the transgenic plants were significantly increased compared with that of WT plants in the present of stresses but not in the absent of stresses (Fig. 9), suggesting that activation of BnaABF2 requires ABA-dependent stress signal transduction. These results are consistent with the early findings that ABA-dependent phosphorylation of ABF2/AREB1 by SnRK2s is necessary for ABF2/AREB1activation of in *Arabidopsis* (Furihata et al. 2006; Yoshida et al. 2010, 2015). Taken together, these data suggest that the enhanced stress tolerance of the *BnaABF2*-expressing transgenic plants can be attributed to expression of downstream genes such as these LEA class genes and ABA-induced stomatal closure (Figs. 6, 9).

Genetic transformation of ABF/AREB transcription factors has been reported as a method for engineering stress-tolerant plants. Overexpression of *ABF2/AREB1* in *Arabidopsis* confers tolerance to drought, high salt, heat and oxidative stresses (Kim et al. 2004b). Additionally, enhanced drought tolerance was found in transgenic *Arabidopsis* plants expressing *ABF3* or *ABF4* (Kang et al. 2002). Moreover, ABF/AREB transcription factors also have been transformed into lettuce, *Agrostis mongolica*, and trifoliate orange. The expression of *ABF/AREB* family members renders increased drought or/and cold/heat stress tolerance to the transgenic plants (Vanjildorj et al. 2005, 2006; Huang et al. 2010). All these successful examples are presented to show the effects of genetic transformation of ABF/AREB transcription factors for engineering stress-tolerant plants. Moreover, BnaABF2 with physiological function has already been cloned from rapeseed in this study and transgenic *Arabidopsis* plants expressing *BnaABF2* were found to be tolerant to drought and salt stresses. Further study on generating stress-tolerant rapeseed plants by genetic transformation of BnaABF2 will be performed soon.

Additionally, the transgenic plants expressing *BnaABF2* showed early flowering phenotype (Fig. 4). This result was consistent with the late flowering phenotype of the *abf1*-*2 areb1 abf3 areb2* and *areb1 abf3 areb2* mutants in *Arabidopsis* (Yoshida et al. 2015). Although some phosphoproteome analyses demonstrated that ABF1, ABF2/AREB1, ABF3 and ABF4/AREB2 are the main substrate transcription factors of SRK2D/SnRK2.2, SRK2E/SnRK2.6 and SRK2I/SnRK2.3 kinases in ABA signalling (Umezawa et al. 2013; Wang et al. 2013), it is noteworthy that the *srk2d/e/i* triple mutants show the early flowering phenotype (Wang et al. 2013). Therefore, ABFs and SRK2D/E/I may participate in a complex network of *Arabidopsis* flowering pathway. Further studies are required to obtain a comprehensive understanding of the flowering mechanism regulated by *BnaABF2* in rapeseed.

## Additional file

**Additional file 1: Table S1.** Primer sequences were used for plasmid constructions in this study. **Table S2.** Primer sequences were used for semi-quantitative and quantitative RT-PCR experiments in this study.

## Abbreviations

ABA: abscisic acid
ABFs: ABRE binding factors
ABRE: ABA-responsive element
AREBs: ABRE binding proteins
bZIP: basic region leucine zipper
CE: coupling element
CPKs: calcium-dependent protein kinases
EST: expressed sequence tag
LEA: late embryogenesis abundant
PP2Cs: 2C protein phosphatases
PYR/PYL/RCARs: pyrabactin resistance 1/PYR1-like receptors
SnRK2 s: SNF1-type kinases
WT: wild-type
