# Supplementary material for: BnaABF2, a bZIP transcription factor from rapeseed (Brassica napus L.), enhances drought and salt tolerance in transgenic Arabidopsis
Source: Bot Stud. 2016 Jun 1;57:12. doi: 10.1186/s40529-016-0127-9 (PMC5432893; doi:10.1186/s40529-016-0127-9)
Supplement: Supplementary file 1 — Additional file 1: Table S1. Primer sequences were used for plasmid constructions in this study. Table S2. Primer sequences were used for semi-quantitative and quantitative RT-PCR experiments in this study. [file 40529_2016_127_MOESM1_ESM.docx]

| **Table S1** Primer sequences were used for plasmids constructions in this study. | |
| --- | --- |
| pUBQ10-BnaABF2 | F: 5’- CGGGATCCATGAATTTCAAGAACAACAAC  AAC-3’ |
|  | R: 5’- TCCCCCGGGTCACCAAGGTCCTGACTCTG  TCC-3’ |
| GFP-BnaABF2 | F: 5’- CGGACTAGTATGAATTTCAAGAACAACAA  CAA-3’ |
|  | R: 5’- CCGCTCGAGTCACCAAGGTCCTGACT-3’ |
| BD-BnaABF2 | F: 5’- CCGGAATTCATGAATTTCAAGAACAACAA  CAA-3’ |
|  | R: 5’- AAACTGCAGTCACCAAGGTCCTGACT-3’ |

| **Table S2** Primer sequences were used for semi-quantitative and quantitative RT-PCR experiments in this study. | |
| --- | --- |
| *ACTIN2* | F: 5’-ATGGCAGACGGTGAGGATATTCA-3’ |
|  | R: 5’-GCCTTTGCAATCCACATCTGTTG-3’ |
| BnaABF2 | F: 5’-TTCAAGAACAACAACAACATGG-3’ |
|  | R: 5’-TGTTCGTCAGAGACTGATCTCC-3’ |
| *β-ACTIN8* | F: 5’-AGTGGTCGTACAACCGGTATTGT-3’ |
|  | R: 5’-GAGGATAGCATGTGGAAGTGAGAA-3’ |
| *RAB18* | F: 5’-CAGCAGCAGTATGACGAGTA-3’ |
|  | R: 5’-CAGTTCCAAAGCCTTCAGTC-3’ |
| *RD29B* | F: 5’-ACTGCTTACGGGCAGAAAGT-3’ |
|  | R: 5’-TTGTTGCGTCTCCTTCACTC-3’ |
| *KIN2* | F:5’-ACCAACAAGAATGCCTTCCA-3’ |
|  | R:5’-ACTGCCGCATCCGATATACT-3’ |
